# The haemodynamic effects of pneumoperitoneum on pulse pressure variation – a prospective, observational study

**DOI:** 10.1007/s10877-025-01300-3

**Published:** 2025-05-05

**Authors:** Henrik Lynge Hovgaard, Simon Tilma Vistisen, Johannes Enevoldsen, Frank Vincenzo de Paoli, Peter Juhl-Olsen

**Affiliations:** 1 Department of Cardiothoracic- and Vascular Surgery, Anaesthesia Section, Aarhus University Hospital, Palle Juul-Jensens Boulevard 99, Aarhus N, 8200 Denmark; 2 Departement of Clinical Medicine, Aarhus University, Palle Juul-Jensens Boulevard 99, Aarhus N, 8200 Denmark; 3 Department of Cardiothoracic and Vascular Surgery, Surgical Section, Aarhus University Hospital, Palle Juul-Jensens Boulevard 99, Aarhus N, 8200 Denmark

**Keywords:** Haemodynamic monitoring, Pulse pressure variation, Stroke volume variation, Pneumoperitoneum

## Abstract

The effects of pneumoperitoneum on dynamic predictors of fluid responsiveness such as pulse pressure variation (PPV) remain uncertain. This uncertainty arises from potentially opposing physiological mechanisms that affect cardiovascular dynamics during conditions with increased intra-abdominal pressure (IAP). Deriving PPV with high precision during induction of pneumoperitoneum may provide new insights into the complex relationship between intra-abdominal pressure changes and PPV. The hypothesis was that PPV derived from a generalised additive model (PPV_GAM_) would increase with the induction of pneumoperitoneum and the associacted increase in IAP. This was a prospective, observational study in patients undergoing oesophagectomy. Before and after induction of pneumoperitoneum, haemodynamic variables including PPV and stroke volume variation (SVV) were recorded with the Hemosphere monitor. PPV_GAM_ was estimated offline from the arterial blood pressure curve. A total of 34 patients were included in the final analysis. PPV_GAM_ increased by a factor of 1.49 (95% CI: 1.25–1.77) as intra-abdominal pressure increased from baseline to 12 mmHg. SVV and PPV from the HemoSphere monitor increased with a factor of 1.25 (95% CI: 1.13–1.39, *p* < 0.001) and 1.14 (95% CI: 1.00–1.29, *p* = 0.048), respectively. PPV derived from a generalised additive model increased approximately 50% from the induction of pneumoperitoneum to an IAP of 12 mmHg. PPV and SVV derived from the Hemosphere monitor also increased signicantly.

**Supplementary Information:**

The online version contains supplementary material available at 10.1007/s10877-025-01300-3.

## Background

Pulse pressure variation (PPV) and stroke volume variation (SVV) are validated predictors of fluid responsiveness during controlled mechanical ventilation and broadly recommended for guiding fluid administration in high-risk surgical procedures [1].

The brief physiological explanation of SVV and PPV is that positive pressure ventilation induces cyclic preload changes to the left ventricle by a number of physiological mechanisms [2]. SVV indicates the resultant variation in stroke volume (SV), while PPV reflects the corresponding pulse pressure (PP) changes. When SVV or PPV exceed 10–15%, the left ventricle is likely operating on the ascending part of the Frank-Starling curve and will increase its output if a fluid bolus is administered. This is termed fluid responsiveness. In patients who are fluid responsive, fluid administration (usually 250–500 mL) increases cardiac output (CO). Correct interpretation of SVV and PPV as dynamic predictors of fluid responsiveness can guide clinicians in the timing of fluid administration and, importantly, in avoiding fluid over-administration. Excessive fluid administration can be detrimental to patient outcome, leading to postoperative complications such as delayed wound healing, cardiopulmonary complications and acute kidney injury causing prolonged hospitalisation [3, 4].

However, SVV and PPV are influenced by multiple factors unrelated to intravascular fluid status, including the intra-abdominal pressure (IAP) [5]. The decrease in IAP during open abdominal surgery lowers SVV [6]. Conversely, during laparoscopy, the increased IAP resulting from pneumoperitoneum alters left ventricular preload and afterload [7] with potential effects on SVV and PPV [8]. As laparoscopic surgeries are adopted widely [9], it is crucial to validate haemodynamic monitoring techniques to ensure accurate fluid management in the context of pneumoperitoneum.

Earlier research on the reliability of PPV and SVV as predictors of fluid responsiveness after induction of pneumoperitoneum has reported conflicting findings [8]. Hence, a more detailed mapping of the interplay between IAP changes and heart-lung interaction is warranted. The study hypothesised that PPV derived from a generalised additive model (PPV_GAM_) [10] would increase with the induction of pneumoperitoneum and the associacted increase in IAP.

## Methods

This was a single-centre, prospective, observational study on a subgroup of patients from a randomised, controlled trial (RCT) [11]. The RCT included five pilot patients that were also eligible for the current study.

The Central Denmark Regional Ethical Committee approved the protocol (case number: 1-10-72-314-21) and all participants gave written and oral informed consent prior to participation.

The study was conducted according to the principles of the Declaration of Helsinki.

### Eligibility criteria

Patients were eligible for inclusion if they were scheduled for elective oesophagectomy at the Department of Cardiothoracic- and Vascular Surgery, Aarhus University Hospital, were 18 years of age or older, and scheduled for laparoscopic surgery as part of the procedure.

Women of childbearing age without a negative pregnancy test were excluded along with patients with a cardiac pacemaker, patients with chronic or paroxysmal atrial fibrillation or patients who did not consent to participation. Additionally, patients with a known left ventricular ejection fraction below 40% or a known tricuspid annular plane systolic excursion of less than 17 mm indicative of right ventricular failure were excluded.

Patients were excluded from final analyses if data quality was compromised by irregular cardiac rhythm or if respiratory rate, tidal volume or positive end-expiratory pressure (PEEP) were changed during abdominal insufflation. Patients were also excluded in cases of excessive noise on the arterial blood pressure (ABP) waveform or if the IAP measured with the insufflation device was missing or deemed unreliable.

### Procedures

#### Induction of anaesthesia

Patients received a thoracic epidural and general anaesthesia was induced with 2–3 mg/kg of propofol, 3–4 ug/kg of fentanyl and 0.6 mg/kg of rocuronium. Anaesthesia was maintained with propofol, fentanyl or remifentanil and epidural bupivacaine 0.25 mg/mL or 0.5 mg/mL.

#### Ventilator settings

Tidal volume was 8 mL/kg ideal body weight (defined as 22 × actual height^2^ (m) regardless of sex) [12] and PEEP was set at 5 cmH_2_O. Respiratory frequency and the fraction of inspired oxygen were at the discretion of the anaesthesiologist in charge of patient care.

#### Surgery and pneumoperitoneum

All included patients underwent a two-stage surgical approach (Ivor Lewis oesophagectomy) starting in a 0-degrees supine position with laparoscopy. Pneumoperitoneum was induced with CO_2_ using a UHI-4 High Flow Insufflation Unit (Olympus Medical Systems, Hamburg, Germany) to an IAP of 12 mmHg.

During induction of pneumoperitoneum, no adjustment in fluid dosage, vasopressor dosage, transducer position or ventilator settings were allowed.

#### Data collection, synchronisation, and analyses

Prior to anaesthesia induction, a radial artery line was inserted and connected to a FloTrac- or Acumen IQ sensor and HemoSphere monitoring platform (Edwards Lifesciences, CA, USA). ECG and ABP waveforms were recorded.

Haemodynamic waveforms were sampled at 125 Hz (e.g. ECG and ABP) and haemodynamic trend variables such as heart rate (HR) and peripheral oxygen saturation (SpO_2_), sampled at 0.5 Hz, were recorded from a Philips IntelliVue MX750 Patient Monitor (Philips, Amsterdam, Netherlands) using VitalRecorder [13] version 1.9.6.0 or later. CO, SVV, PPV (PPV_HemoSphere_) were estimated by the HemoSphere monitor every 20 s and also collected with VitalRecorder.

SV and SVR were unsuccessfully exported from the HemoSphere monitor to VitalRecorder and these variables were calculated retrospectively.

Airway pressures were automatically recorded in the local patient data monitoring system (PDM, Systematic Healthcare, Denmark) at a frequency of 1 min^− 1^.

The insufflation unit did not offer data export and during inflations, the measured IAP was stored on video, including the computer time for the VitalRecorder laptop, allowing offline synchronisation of the abdominal inflation procedure with haemodynamic monitoring variables. The baseline time point and the time points of reaching an IAP of 2, 4, 6, 8, 10, 12 mmHg during abdominal insufflation were determined offline.

The ABP waveform was filtered with a first order Butterworth filter and a cut-off frequency of 15 Hz. The respiratory frequency was automatically detected by identifying the dominant frequency component in the [0.1–0.5 Hz] range of the Fourier transform in the ABP signal. A previously published custom-made algorithm [10] that identifies each heartbeat in the filtered ABP waveform was applied. The algorithm further determines the PP and identifies possible ectopic and post-ectopic beats for exclusion. Potential ectopic/post-ectopic beats were confirmed or occasionally corrected after visual inspection. For the baseline and IAP = 12 mmHg time points, the PPV_GAM_ was calculated using a GAM approach. In brief, the GAM offers high-accuracy PPV estimates [14]. PPV_GAM_ was derived from 30-second time windows where the last sample corresponded to the determined time points. PPV_GAM_ was calculated as the maximal minus the minimal value of the GAM-estimated respiratory effect on PP, divided by the average PP, also estimated by the model.

The complete details and the open-source code for these processing steps are described elsewhere [10, 14].

In this study, we present PPV_GAM_ along with other relevant measurements at baseline (median of measurements 30 s before the start of insufflation) and at an IAP of 12 mmHg (median of the last 30 s before the end of insufflation).

### Endpoints

The primary endpoint was the change in PPV_GAM_ from baseline IAP to an IAP of 12 mmHg.

Secondary endpoints included changes in other haemodynamic variables available from the HemoSphere (SV, CO, SVR, SVV, PPV_HemoSphere_) and Philips monitor (HR, ABPs and CVP).

### Statistical analyses

Means with standard deviations (SD) or medians with interquartile ranges (IQR) are provided for continuous variables. For categorical variables, counts and percentages are presented. The normality assumption was assessed by inspection of density- and QQ-plots. Further, the presence of heteroscedasticity of insufflation-induced absolute changes in haemodynamic variables was assessed with Bland-Altman plots.

In case of significant heteroscledacity or right skewed distributions on the absolute scale, we estimated the effect of pneumoperitoneum as the relative change from baseline to the end of insufflation using a paired t-test of log-transformed estimates. This was the case for PPV_GAM_, PPV_HemoSphere_ and SVV.

In the case of normally distributed variables and no significant heteroscedasticity, the absolute changes from baseline to end of insufflation and their certainties are presented.

To assess agreement between PPV_GAM_ and PPV_HemoSphere_ Bland-Altman analyses were performed at baseline and at an IAP of 12 mmHg.

We did not have relevant previous PPV_GAM_ data during pneumoperitoneum to perform a meaningful power calculation and the cohort is a convenience sample.

Two-sided P-values of < 0.05 defined statistical significance.

Data analysis was performed in R (R Foundation for Statistical Computing, Vienna, Austria), RStudio [15] Version 2024.12.0 + 467 (2024.12.0 + 467) using the packages dplyr, ggplot2, ggpubr, gtsummary, mgcv, edfreader, lubridate, and gratia [16]. All packages were updated before running data analyses on the 29.11.2024.

## Results

**Fig. 1:**
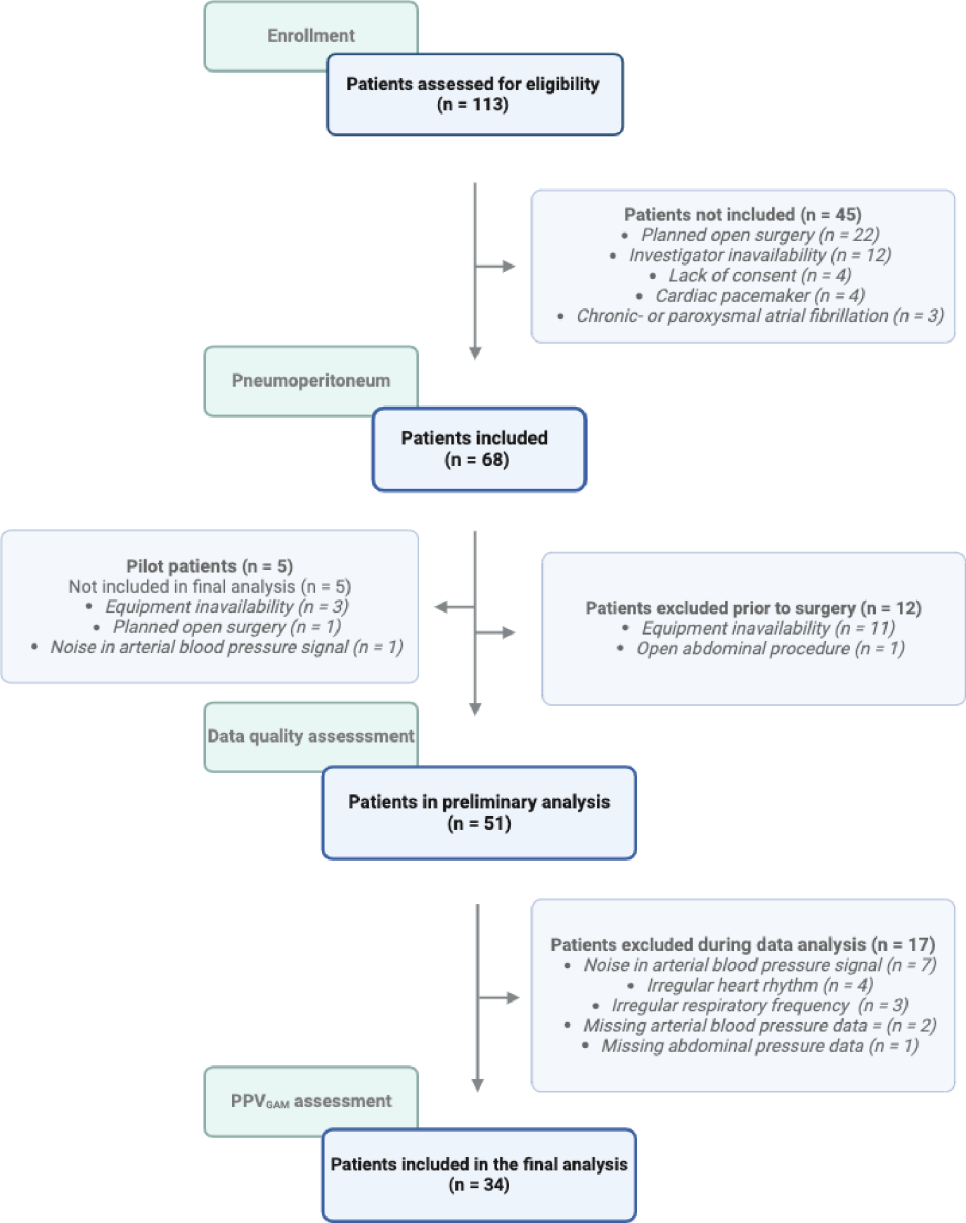
Description of patient eligibility, inclusion and exclusion. PPV_GAM_: Pulse pressure variation derived with the generalised additive model

The study included 68 patients of whom 34 patients were excluded from analyses due to open abdominal procedure, equipment inavailability or poor data quality. The final analysis included 34 patients (Fig. 1; Table 1). Of these, 28 (82%) were equipped with the FloTrac or Acument IQ sensor connedted to the HemoSphere monitor. All patients had reliable PPVw_GAM_ measurements and 31 (91%) patients had PPV_GAM_ < 10% at baseline.

**Table 1:** Patient characteristics

Variable	*n* = 34
**Age (years)**	68 (9)
**Male gender**	26 (76%)
**Body mass index (kg/m** ^**2**^ **)**	26.9 (4.6)
**Ideal body weight (kg)**	69.2 (9.1)
**FEV1 (% of expected**, ***n* = 30)**	94 (19)
**Hypertension diagnosis**	17 (50%)
**Preoperative smoking status**	
Never	28 (82%)
Stopped within eight weeks before surgery	5 (15%)
Current smoker	1 (2.9%)
**Preoperative chemotherapy**	23 (68%)
**Preoperative radiotherapy**	23 (68%)
**Mean night-time MAP (mmHg**, ***n* = 32)**	70 [64, 77]
**ASA-class III**	34 (100%)
**Charlson comorbidity index**	5 [4, 5]

Baseline characteristics presented as mean (standard deviation), median [interquartile range] or number (%). Missing data is indicated in the table and was not imputed

Mean night-time MAP: The mean of the patient’s three lowest MAP values measured hourly between 10PM and 06AM before surgery

FEV1: Forced expiratory ventilation in one second, MAP: Mean arterial pressure, ASA: American Society of Anesthesiologists physical status classification

**Fig. 2:**
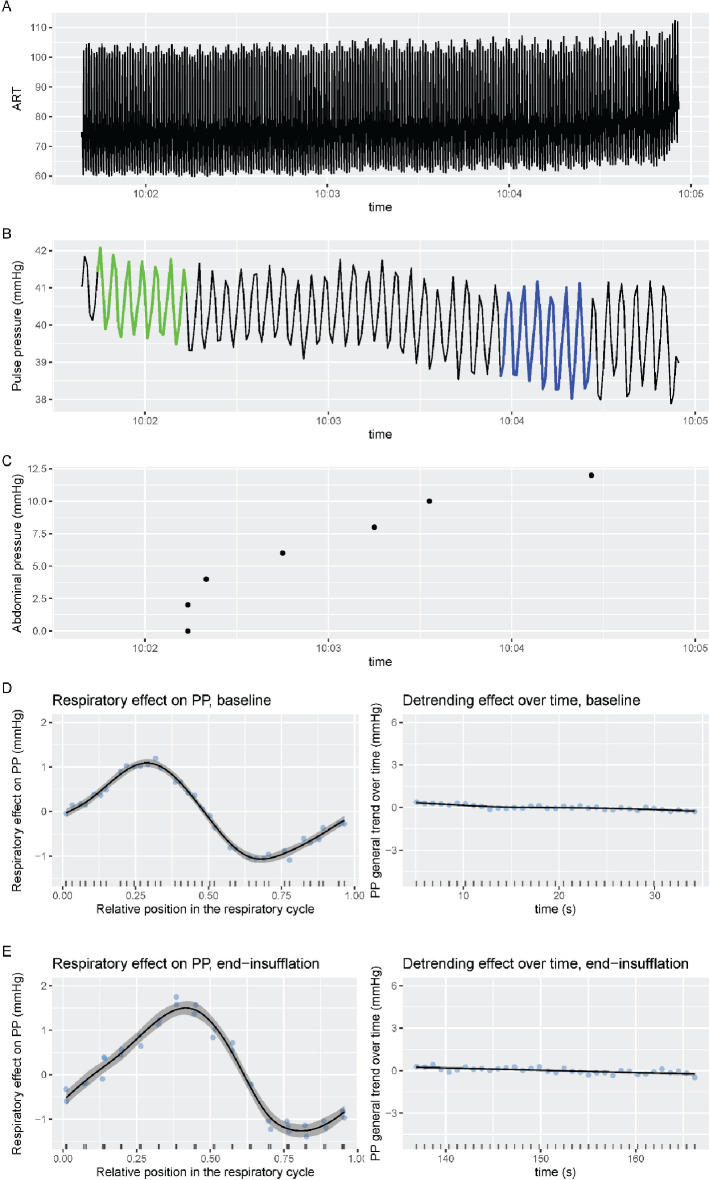
Example of how general additive model (GAM)-modelling was performed in one patient. Panel **A**: The arterial blood pressure curve over time. Panel **B**: The pulse pressures derived from panel **A**. Each cyclic swing in pulse pressure represents one respiratory cycle. Green and blue segments are the sections undergoing GAM-modelling to derive PPV before and at the end of insufflation. Panel **C**: The intra-abdominal pressure time stamps (0 mmHg corresponds to baseline, initiation of insufflation). Panel **D**: The timing of the heartbeat in the respiratory cycle (left) and the effect of time passed (right) and their effect on pulse pressure before induction of pneumoperitoneum. Panel E: Represent the same calculations as panel D, but for measurements at the end of pneumoperitoneum induction at an intra-abdominal pressure of 12 mmHg ART: Arterial blood pressure, PP: pulse pressure

The lowpass-filtered ABP waveform during the entire pneumoperitoneum insufflation process, derived PP time series, and the GAM-fitted respiratory effect in PP at baseline and IAP of 12 mmHg are shown in Fig. 2 for a representative patient.

**Fig. 3:**
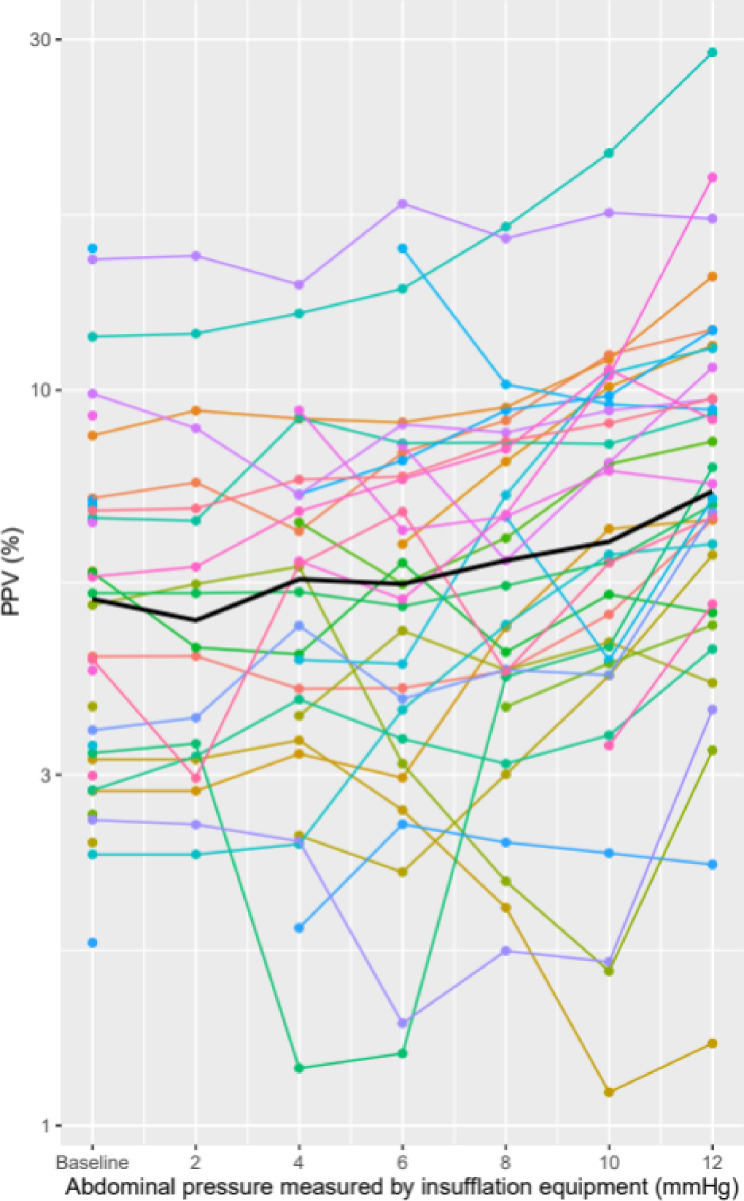
Spaghetti plot of generalised additive model (GAM)-derived pulse pressure variation (PPV) and its change on the logarithmic scale with different intra-abdominal pressures. Black line: median GAM-derived PPV

From baseline IAP to an IAP of 12 mmHg, PPV_GAM_ increased by a factor 1.49 (95% CI: 1.25–1.77, *p* < 0.001, Table 2; Fig. 3). The corresponding GAM modulated PP curves for all 34 patients are shown in Supplemental Material Fig. 1. From crude visual inspections, PPV_GAM_ was modestly affected in the first half of the insufflation, and generally increased most in the last half of insufflation. PPV_HemoSphere_ increased by a factor of 1.14 (95% CI: 1.00–1.29, *p* = 0.048) and SVV increased by a factor of 1.25 (95% CI: 1.13–1.39, *p* < 0.001) with insufflation.

**Table 2:** Summary of all measurements at baseline and at an IAP of 12 mmhg

Measurement	Pre-Insufflation	Post-Insufflation	Difference	95% Confidence Interval		*P*-value
				Lower	Upper	
Peripheral oxygen saturation (SpO_2_, %)	99 (1)	99 (2)	0	−0	0	0.28
Peak inspiratory pressure (cmH_2_O)	17 (4)	21 (4)	4	4	5	< 0.001
Inspiratory plateau pressure (cmH_2_O)	16 (4)	21 (3)	4	4	5	< 0.001
End expiratory airway pressure (cmH_2_O)	5 (1)	5 (1)	0	Na	Na	1.00
Heart rate (beats/min)	63 (12)	68 (11)	4	3	6	< 0.001
Diastolic blood pressure (mmHg)	59 (8)	66 (9)	6	4	9	< 0.001
Systolic blood pressure (mmHg)	113 (15)	121 (19)	7	2	13	0.012
Mean arterial pressure (mmHg)	78 (10)	86 (13)	8	4	12	< 0.001
Central venous pressure (mmHg)	12 (6)	14 (6)	2	1	3	0.001
Stroke volume (mL)	79 (27)	72 (25)	−7	−11	−3	< 0.001
Cardiac output (L/min)	4.8 (1.6)	4.7 (1.5)	−0.1	−0.4	0.2	0.38
Systemic vascular resistance (dyn·s/cm^5)	1210 (334)	1343 (451)	133	23	244	0.020
Stroke volume variation (%)*	8 (3)	10 (4)	1.25	1.13	1.39	< 0.001
PPV from the HemoSphere monitor (%)*	8 (2)	9 (4)	1.14	1.00	1.29	0.048
PPV (GAM-modulated, %)*	6 (3)	9 (5)	1.49	1.25	1.77	< 0.001

Haemodynamic variables at baseline (pre-insufflation) and at an intra-abdominal pressure of 12 mmHg (post-insufflation). Measurements are presented as mean (standard deviation). *Data was log-transformed due to heteroscedasticity. Hence, the estimates and the confidence intervals are presented on the relative scale. GAM: Generalised additive model, PPV: Pulse pressure variation. IAP: Intra-abdominal pressure, SpO_2_: Peripheral oxygen saturation

Seven patients showed an increase in PPV_GAM_ from < 10% before induction of pneumoperitoneum to ≥ 10% at an IAP of 12 mmHg. For PPV_HemoSphere,_ analogous increases were seen in five patients.

The mean biases of the absolute PPV values were 0.08% (95% CI: -1.08 to 1.23) at baseline and 1.62% (95% CI: 0.66 to 2.57) at an IAP of 12 mmHg. The 95% limits of agreement were − 3.43–6.67% at baseline and − 6.04–6.19% at an IAP of 12 mmHg. Bland-Altman plots are presented in supplementary material 1.

The physiological changes observed from baseline IAP to 12 mmHg are given in detail in Table 2.

From baseline to an IAP of 12 mmHg, mean systemic ABP and CVP increased 8 mmHg (95% CI: 4–12, *p* < 0.001) and 2 mmHg (95% CI: 1–3, *p* < 0.001), respectively.

## Discussion

Induction of pneumoperitoneum increased PPV_GAM_ by approximately 50%. PPV_HemoSphere_ and SVV also increased significantly. Systemic blood pressures and HR increased, whereas CO remained unchanged, SV decreased and SVR increased.

No previous studies have isolated and specified the effects of increases in IAP on PPV to the level of detail achieved in the present study. However, some studies have reported how PPV and SVV are affected by pneumoperitoneum after establishing a new haemodynamic steady state [17–19].

Our findings align with a study by Chin et al. [17] who demonstrated that when IAP increased to 20 mmHg, SV decreased while both PPV and SVV nearly doubled.

Similary, Ghoundiwal et al. [18] reported PPV and SVV values at baseline and after five minutes of haemodynamic stabilisation at an IAP of 12 mmHg. Under these conditions, no significant differences in PPV or SVV were observed following the increase in IAP.

Høiseth et al. [19] also reported no significant changes in PPV and SVV after reaching an IAP of 12 mmHg. However, significant positional changes preceded pneumoperitoneum in that study [19] and this may have influenced the results.

Overall, previous studies found diverging effects of IAP increases on PPV and SVV under conditions comparable to the present study. Differences in study methodologies, including settings and measurement approaches, likely contribute to the observed discrepancies. Notably, none of the prior studies tracked changes in PP during IAP increases.These discrepancies, along with small sample sizes in earlier research may explain the variability in results.

An additional factor contributing to the variation between this study and previous research is the inherent uncertainty of PPV estimates [20]. A confirmed source of variability lies in the number of PPs and respiratory cycles used to estimate PPV. This sampling variability may lead to different PPV estimates derived from the same ABP curve. Increasing the number of respiratory cycles in the analysis generally leads to higher PPV estimates [20]. This consideration is particularly relevant when comparing the present results to those of prior studies. Previous research [17–19] reported PPV values obtained using the Philips Intellivue software, whereas this study utilised PPV measurements from the HemoSphere platform and publically available algorithms (PPV_GAM_) [10]. Furthermore, our offline visual inspection of PP detection and sinus rhythm could reduce the noise level in comparison with the automated detection in the two monitors.

### Physiological considerations

The effects of increasing IAP on PPV involve complex and, at times, opposing physiological mechanisms that affect both the systemic- and pulmonary circulations [21].

**Fig. 4:**
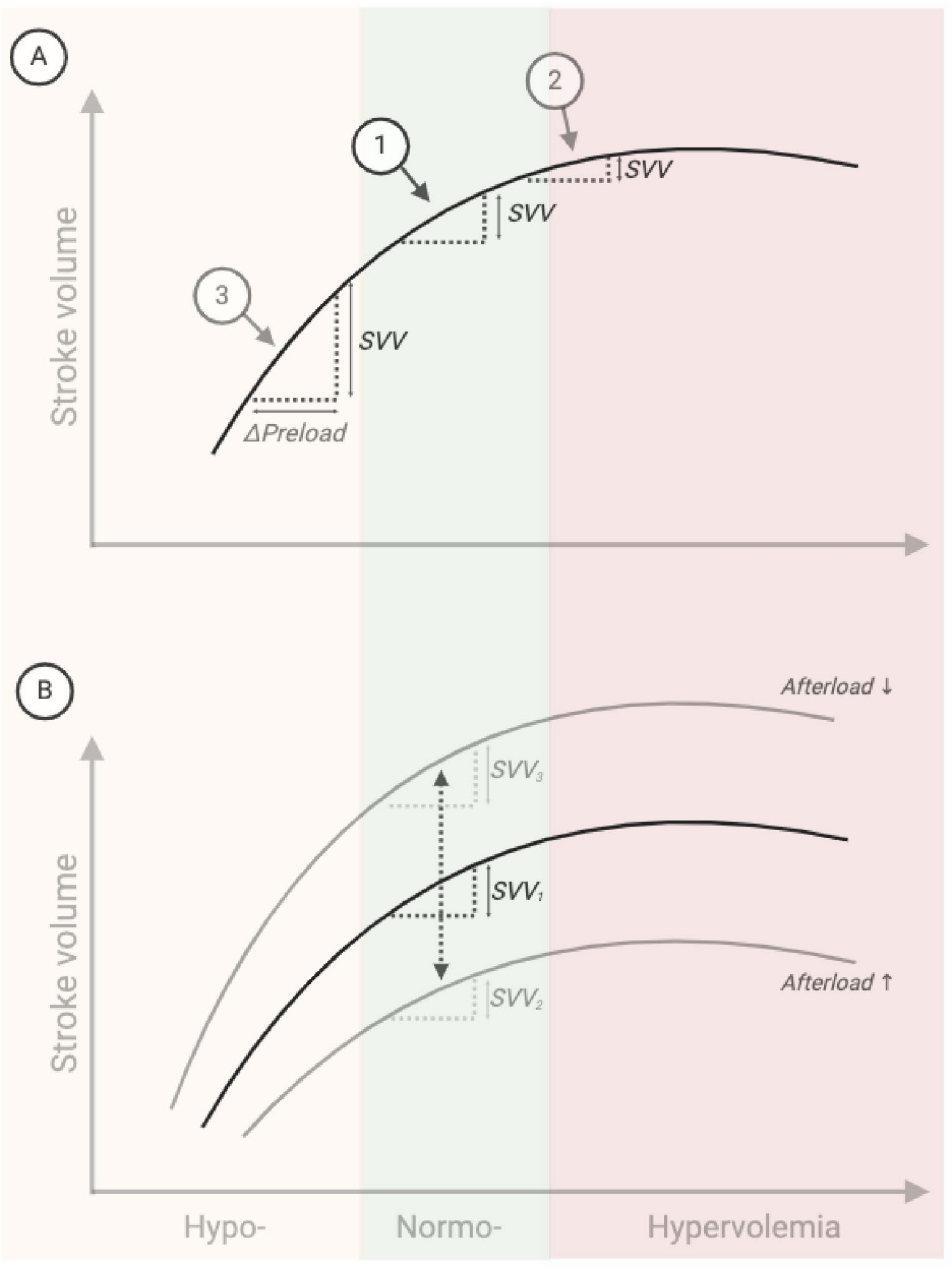
Illustrations of Frank Starling curves and immediate effects of intra-abdominal pressure on the left ventricle. (**A**) Left and right-wards shifts in operating position, slope and their influence on SVV. ① baseline ② increased preload ③ decreased preload. (**B**) Downward and upward shifts of the Frank Starling curve with increased afterload, reduced contractility or vice versa. SVV_1_: baseline, SVV_2_: increased afterload, SVV_3_: decreased afterload. ΔPreload: Preload variation during positive pressure ventilation, SVV: Stroke voume variation

In the abdominal compartment, elevated IAP reduces venous capacitance and increases the pressure gradient between the abdominal veins and the right atrium. The augmented pressure gradient enhances venous return and thereby right ventricular preload. In isolation, this effect shifts the operating point on the Frank-Starling curve to the right, leading to a reduction in the PPV (Fig. 4A). Conversely, higher IAP also reduces the transmural venous pressure of the abdominal vessels and increases resistance to venous outflow. Increased venous resistance reduces venous flow and right ventricular preload and will thus tend to increase PPV by causing a leftward shift of the operating point on the Frank-Starling curve (Fig. 4A).

The upward displacement of the diaphragm during pneumoperitoneum compresses the lungs, reducing lung compliance. This results in an increase in ventilatory driving pressure (inspiratory plateau pressure minus end-expiratory pressure) while maintaining constant tidal volumes. These findings align with the results of this study (Table 2), as evidenced by a slight increase in mean airway pressure. The haemodynamic effects of increased mean airway pressure likely include increased pulmonary vascular resistance, reduced right ventricular SV, and lower left ventricular filling. Lower left ventricular filling causes a leftward shift of the operating point on the Frank-Starling curve (Fig. 4A) leading to an increase in PPV. Regardless of the the slightly increased mean airway pressure and shift on the Frank-Starling curve operating point, the increased driving pressure may itself cause PPV to increase, because it is a likely driver of the preload changes and thus associated with the magnitude of PPV [2, 14].

In the systemic circulation, higher IAP increases absolute blood pressures. This increase may result from the external pressure applied directly on abdominal arteries and the physiological effects of absorbed CO_2_ [22]. Although, CO_2_ is known to have vasodilatory properties, acute increases in arterial CO_2_ can induce systemic arterial constriction through a chemoreceptor-mediated sympathetic response [23]. The direct mechanical effect of IAP on systemic blood pressure may be amplified by the absorption of insufflated CO_2_. The combined effects increase left ventricular afterload and lower SVV (Fig. 4B).

The net effect of IAP on SVV and PPV likely results from a combination of preload and afterload alterations.

The observed reduction in SV in this study suggests that pneumoperitoneum induces a leftward shift of the operating point on the Frank-Starling curve and/or a downward displacement of the Frank-Starling curve. Alternatively, this reduction in SV may reflect a physiological reflex to the increase in HR, or vice versa, maintaining CO constant. Additionally, the reduction in SV may partially result from the increased afterload associated with elevated IAP.

### Potential clinical implications

A PPV greater than 10% is predictive of fluid responsiveness [24]. Induction of pneumoperitoneum caused a shift across this PPV threshold in one-fifth of the patients. In a clinical setting, the combination of increasing PPV, elevated HR and reduced SV likely prompts fluid administration. Even if the increase in PPV is predominantly caused by a leftward shift shift of on the Frank-Starling curve operating poing, indicative of central hypolaemia, the haemodynamic effects of pneumoperitoneum will be nullified during the subsequent abdominal desufflation. Hence, any fluid administered due to the haemodynamic changes caused by pneumoperitoneum may stabilise SV during the insufflation period but likely contributes to venous stasis after desufflation. Awareness of this trade-off is clinically relevant. If PPV increases predominantly because of increased driving pressure, it may be worth to consider a new, individual baseline level for PPV to guide fluid therapy during the insufflation period.

### Limitations

Pneumoperitoneum elicits pain, which may contribute to the haemodynamic changes observed in this study. Pain stimuli may increase HR, blood pressures, SV and CO [25]. Although we cannot be sure that general anaesthesia fully alleviated pain perception, the decrease in SV and unchanged CO observed suggests that any response to a pain stimulus was minimal.

Tidal volume and respiratory frequency were kept constant during abdominal insufflation and therefore PaCO_2_ was not controlled. CO_2_ absorbed from the abdominal cavity may have increased PaCO_2_ causing haemodynamic changes.

Hypercarbia of > 60 mmHg or 8 kPa can produce similar haemodynamic effects as seen in this study [21, 26]. CO_2_ absorption is detectable as an average increase in PaCO_2_ of 10 mmHg or 1.3 kPa after five minutes of pneumoperitoneum [21]. However, in this study, the maximal duration of abdominal insufflation from baseline to an IAP of 12 mmHg was six minutes which was likely insufficient to allow CO_2_ absorption to produce significant haemodynamic changes. The brief period where measurements were undertaken also reduces the possibility of observing physiological mechanisms which are not immediate, including the the Anrep effect.

As abdominal desufflation occurs passively with retraction of surgical instruments, simultaneous measurements of IAP cannot be performed which precludes a comparison of the reversibility of changes with the changes that occurred during insufflation.

Further, our results are restricted to patients with relatively low PPVs, as 31/34 patients had PPV_GAM_ under 10% at baseline.

Finally, CO values were obtained from the uncalibrated 4th -generation FloTrac software. These CO-estimates are subject to measurement bias [27] and, importantly, non-perfect trending ability [27, 28]. Any inaccuracy in CO estimation would have directly influenced the calculations of SV and SVR.

## Conclusion

GAM-modulated PPV increased approximately 50% from the induction of pneumoperitoneum to an IAP of 12 mmHg. PPV and SVV derived from the Hemosphere monitor also increased signicantly.

The study provides a comprehensive exploration of the haemodynamic effects of increases in IAP on established predictors of fluid responsiveness and other haemodynamic measurements, offering insights into the physiological complexities introduced by pneumoperitoneum.

## Electronic supplementary material

Below is the link to the electronic supplementary material.

Supplementary Material 1

Supplementary Material 2

## Data Availability

No datasets were generated or analysed during the current study.
