# Supplementary figures and images for: The haemodynamic effects of pneumoperitoneum on pulse pressure variation – a prospective, observational study
Source: J Clin Monit Comput. 2025 May 5;39(5):863–73. doi: 10.1007/s10877-025-01300-3 (PMC12474645; doi:10.1007/s10877-025-01300-3)

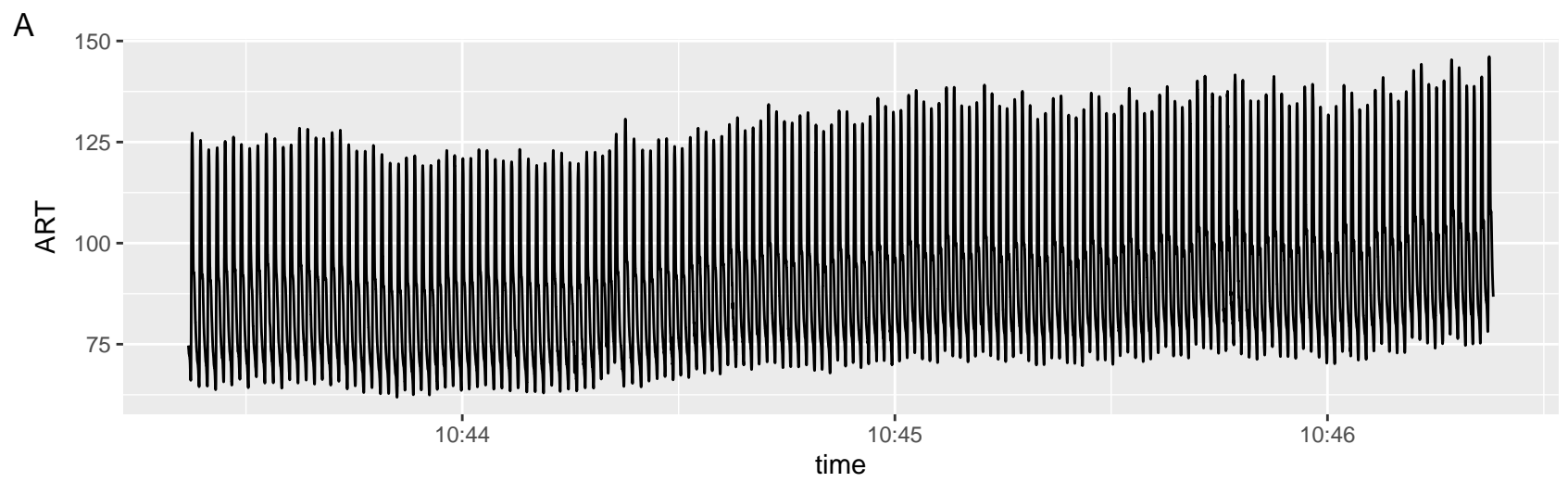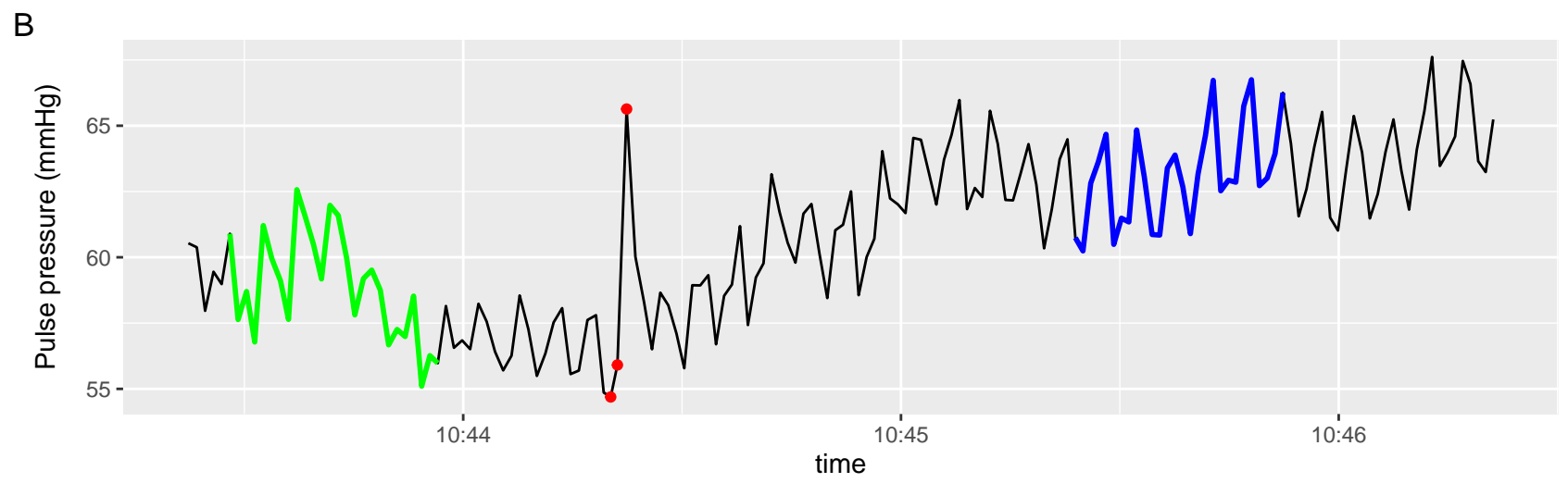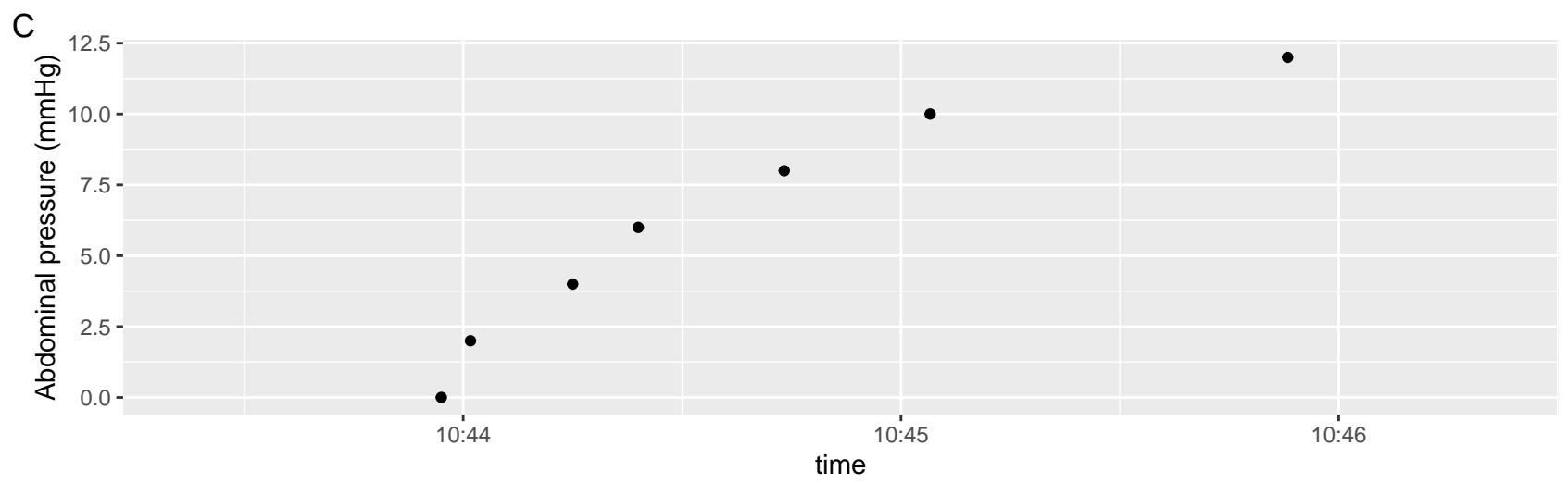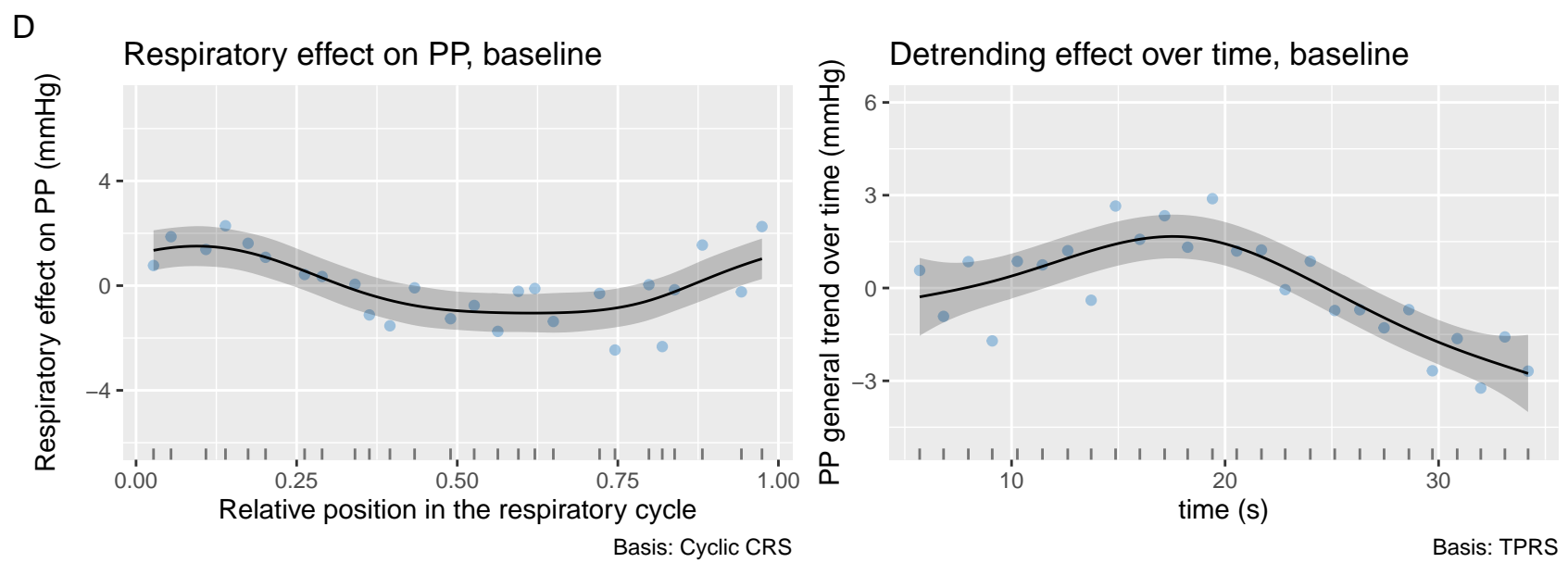

Supplement: Supplementary file 1 — Supplementary Material 1 [file 10877_2025_1300_MOESM1_ESM.pdf]
