# Supplementary material for: The haemodynamic effects of pneumoperitoneum on pulse pressure variation – a prospective, observational study
Source: J Clin Monit Comput. 2025 May 5;39(5):863–73. doi: 10.1007/s10877-025-01300-3 (PMC12474645; doi:10.1007/s10877-025-01300-3)
Supplement: Supplementary file 2 — Supplementary Material 2 [file 10877_2025_1300_MOESM2_ESM.docx]

# Supplementary figure 1: GAM modelling for all patients

*** Figure uploaded separately ***

*Supplementary figure 1: General additive model (GAM) for all patients*

*Panel A: The arterial blood pressure curve over time.*

*Panel B: The pulse pressures derived from panel A. Each cyclic swing in pulse pressure represents one respiratory cycle. Green and blue segments are the sections undergoing GAM-modelling to derive PPV before and at the end of insufflation.*

*Panel C: The intra-abdominal pressure time stamps (0 mmHg corresponds to baseline, initiation of insufflation).*

*Panel D: The timing of the heartbeat in the respiratory cycle (left) and the effect of time passed (right) and their effect on pulse pressure before induction of pneumoperitoneum.*

*Panel E: Represent the same calculations as panel D, but for measurements at the end of pneumoperitoneum induction at an intra-abdominal pressure of 12 mmHg.*

*ART: Arterial blood pressure, PP: pulse pressure*
